# Enhanced Training Benefits of Video Recording Surgery With Automated Hand Motion Analysis

**DOI:** 10.1007/s00268-020-05916-1

**Published:** 2021-01-03

**Authors:** Colin F. Mackenzie, Shiming Yang, Evan Garofalo, Peter Fu-Ming Hu, Darcy Watts, Rajan Patel, Adam Puche, George Hagegeorge, Valerie Shalin, Kristy Pugh, Guinevere Granite, Lynn G. Stansbury, Stacy Shackelford, Samuel Tisherman

**Affiliations:** 1 School of Medicine (UMDSOM) Shock Trauma Anesthesiology Research Center, University of Maryland, 11 S Paca St, Suite LL-01, Baltimore, MD 21201 USA; 2 Department of Basic Sciences, University of Arizona School of Medicine, Tucson, AZ 85724 USA; 3 Department of Surgery, UMDSOM, Baltimore, MD 21201 USA; 4 Anatomy and Neurobiology, UMDSOM, Baltimore, MD 21201 USA; 5 Department of Psychology, Wright State University, Dayton, OH 45435 USA; 6 Department of Surgery, Uniformed Services University of the Health Sciences, Bethesda, MD 20814 USA; 7 Harborview Injury Prevention and Research Center, University of Washington, Seattle, USA; 8 Joint Trauma System, Defense Health Agency Combat Support, Falls Church, USA

## Abstract

**Background:**

Hand motion analysis by video recording during surgery has potential for evaluation of surgical performance. The aim was to identify how technical skill during open surgery can be measured unobtrusively by video recording during a surgical procedure. We hypothesized that procedural-step timing, hand movements, instrument use and Shannon entropy differ with expertise and training and are concordant with a performance-based validated individual procedure score.

**Methods:**

Surgeon and non-surgeon participants with varying training and levels of expertise were video recorded performing axillary artery exposure and control (AA) on un-preserved cadavers. Color-coded gloves permitted motion-tracking and automated extraction of entropy data from recordings. Timing and instrument-use metrics were obtained through observational video reviews. Shannon entropy measured speed, acceleration and direction by computer-vision algorithms. Findings were compared with individual procedure score for AA performance

**Results:**

Experts had lowest entropy values, idle time, active time and shorter time to divide pectoralis minor, using fewer instruments. Residents improved with training, without reaching expert levels, and showed deterioration 12–18 months later. Individual procedure scores mirrored these results. Non-surgeons differed substantially.

**Conclusions:**

Hand motion entropy and timing metrics discriminate levels of surgical skill and training, and these findings are congruent with individual procedure score evaluations. These measures can be collected using consumer-level cameras and analyzed automatically with free software. Hand motion with video timing data may have widespread application to evaluate resident performance and can contribute to the range of evaluation and testing modalities available to educators, training course designers and surgical quality assurance programs.

**Supplementary Information:**

The online version of this article (10.1007/s00268-020-05916-1) contains supplementary material, which is available to authorized users.

## Introduction

Written or oral examination performances can be unreliable indicators of the real-world technical performance of surgeons. Current assessment of technical skills is based on subjective opinions of senior colleagues [1, 2]. Surgical residents’ technical skill is typically evaluated from observations by experienced mentors during training; however, this process is time-consuming, labor-intensive and may include evaluator biases. Three technical performance evaluations are currently validated, the most often used is the objective structured assessment of technical skills (OSATS) [3]. Procedure-based assessments (PBA) and direct observation of practical skills (DOPS) are integrated within the Intercollegiate Surgical Curriculum Programme online platform and aim to assess trainees’ performance in practical surgical skills [4]. The individual procedure score (IPS) has been validated for vascular hemorrhage control and non-vascular trauma procedures [5–8]. OSATS, PBA and IPS were found to identify surgeons, at all levels of seniority, who are in need of remediation of technical skills for open surgery [3–5]; however, all these evaluations require significant resources and expense.

Video recording of bariatric surgery [9] and left colectomy laparoscopic procedures [10] linked adverse patient outcomes of complications, re-operation and death to differences in technical skills using OSATS evaluations. Video recording using IPS evaluation showed low IPS can predict which surgeons among trainees, practicing surgeons and experts will make critical errors when performing vascular control procedures [5, 8]. Video recording of open surgical procedures in conjunction with hand motion analysis has potential as an unbiased and cost-effective alternative to OSATS, PBA and IPS evaluation of surgeon technical performance. Such video assessment would assist teaching, enable timing of procedural steps, allowing technical skill evaluations to be integrated into residency training. Trainee surgeons could obtain immediate feedback to improve procedural skills and minimize the reinforcement of errors [11]. The elements of manual dexterity on which surgical skill depends have been increasingly well documented over the last decade and are related to levels of experience [12–24]. However, many studies of surgeon hand motion rely on synthetic models or partial tasks [14, 19, 20] to simplify the analysis or focus on endoscopic/laparoscopic/robotic procedures [15, 17, 18, 22] where the surgeons hands move through a limited range of motions around a fulcrum. Few studies evaluate hand movements occurring during open surgical procedures because these procedures vary widely, requiring assessment methods that allow for freedom of hand and instrument movement. Moreover, ideally, these methods should be sensor-free to avoid interference with hand motion and surgical performance [12, 16, 21]. Adoption of electromagnetic [14] optical tracking [19] and sensors attached to hands to quantitate movements has failed due to complexity or technical difficulty.

The combination of kinematic data collection and analysis, and video surgical gesture-recognition has potential to address these requirements [12, 16, 24]. The entropy of a random variable such as hand motion measures the uncertainty of the chaotic movements. Shannon joint entropy has been used to summarize the systematic information conveyed by bimanual hand movements, using computer vision (CV) algorithms derived from measurement of frequency, direction and speed of movement changes [12, 16, 24]. Entropy should decrease with hand motion efficiency and has been used to analyze hand movements in training models of suturing [12] but not to evaluate open surgical procedures. Our aim in the work reported here was to identify how performance of technical skill during open surgery could be measured unobtrusively by video recording during a surgical procedure on human cadavers. We hypothesized that entropy of surgeon hand motion is congruent with a validated measure of surgeon performance and detects differences between experts, resident surgeons and non-surgeons.

## Materials and methods

Axillary artery exposure and proximal control procedures were performed in the State Anatomy Board laboratories at the University of Maryland in Baltimore as part of a study to validate benefits of the Advanced Surgical Skills for Exposure in Trauma course (ASSET) [25]. The cadaver laboratories were equipped with consumer-grade video cameras (Nikon d600) above each table. Shannon joint entropy analysis was used to quantify and summarize bimanual motion [24, 26] captured by 50 frames per second (fps) video using a 50–80 degrees unobstructed field of view that included the incision and operator’s hands.

Variability and ability to discrimination expertise among participants were achieved by testing participants with known differences in skill performing the AA procedure: Two experienced (more than 20 years) attending trauma surgeons, surgical residents (one third-year, one fifth-year) before and immediately after training in AA procedure and 12–18 months later, and two Ph.D. demonstrator/anatomists (anatomically knowledgeable, but inexperienced clinical surgery operators).

Video was reviewed frame-by-frame using *VirtualDub* version 1.10.4 (http://www.virtualdub.org/). Eight metrics were gathered by trained observers of AA video recordings: (1) total time (skin incision to passage of vessel loop); (2) total idle time [27]; (3) total active time; (4) time from skin incision to division of pectoralis minor; (5) number of times instruments changed; (6) blunt dissection time; (7) sharp dissection time; (8) type and duration of instruments used. Start and stop times, active and idle times associated with blunt dissection, and instrument use were recorded from skin incision to passage of the proximal AA vessel loop. Start and stop times were recorded when a hand or surgical instrument made contact with or left contact with the cadaver. Idle time was determined by summation of the time instruments spent outside the surgical incision. Active time was calculated as the duration of the activity of either or both hands while using instruments or dissecting bluntly. Ratios of active to idle time and sharp to blunt dissection were also calculated. The entropy measures were compared with the individual procedure score for AA procedure collected by co-located trained evaluators present during the AA procedures. Individual procedure score evaluations used a standardized script including checklists and global rating scales as previously described [5–8]. Measurement of the individual procedure score for AA by analysis of video collected during the evaluation took about five times the duration of the video recorded procedure.

### Computer vision algorithm feature extraction and quantification

**Fig. 1:**
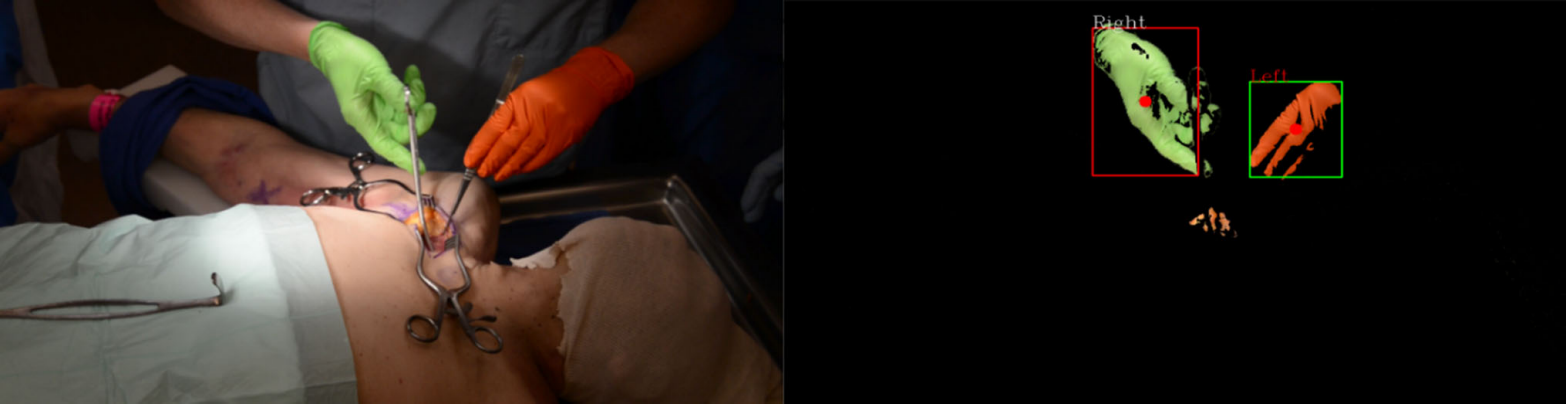
The centers of the minimum rectangular boxes defined by the glove colors (shown as red dot) were calculated for each video frame to define the position of the hand. Shannon joint entropy was used to calculate speed (pixels/s), acceleration (change in speed/s) and change of direction (degree) with a resolution of 1 s

Sensor-free hand motion feature extraction by computer vision algorithms was accomplished using color-coded surgical gloves: green, dominant hand; orange, non-dominant hand. Left and right hand position for each frame was extracted using computer vision software to detect glove colors (Fig. 1). The change of direction θ was quantified by the angle formed by two consecutive movement directions. Joint entropy was calculated as: $H\left(X,Y\right)= -\sum p\left(x,y\right) {log}_{2}(x,y)$, where* X*,* Y* denote the corresponding measurements of dominant and non-dominant hand motion. Entropy measures (speed = pixels/second; acceleration = change in speed/second; directional change = degrees) were compared between operators. Entropy data could be calculated in near real time with a basic computer (Windows 7 (64 bit) machine with 16 Gigabyte memory, intel i5 core 1.3 GHz).

## Results

As shown in Table 1, experts had the shortest times for all time measures, the highest ratio of active to idle times and the fewest instrument changes. Pre-training residents had highest total time, active time, ratio of sharp to blunt dissection and time to pectoralis minor and greatest number of instrument changes. In general, timing and procedural metrics for residents were best immediately after training or when re-evaluated 12 or 18 months later in comparison with pre-training values. Pre-training residents had the longest total time > 1200 s, because neither divided pectoralis minor, a key landmark anterior to the axillary artery, and both failed to expose and encircle the artery with a vessel loop within the 20 min time limit allowed for the procedure. These video analysis metrics were in concordance with individual procedure score changes recorded for the same residents at pre- and post-ASSET and skills-retention evaluations.

**Table 1:** Metrics derived from observational video analysis and computationally derived hand motion entropy

	Anatomists *n* = 2	Experts *n* = 2	Residents pre-training *n* = 2	Residents post-training *n* = 2	Residents retention *n* = 2
Total time in seconds (range)	1030 (815–1246)	315 (303–328)	Both > 1200	619 (328–911)	461 (413–509)
Mean incision time to pectoralis minor	99	64	842	120	120
Active time (range)	968 (785–1152)	307 (294–320)	> 1200	589 (308–871)	425 (391–460)
Idle time as a percent of active	5.6	2.9	5.1	5.1	8.3
Active/idle time ratio	15:1	33:1	19: 1	20:1	12:1
Number of instrument changes (range)	67 (45–89)	23 (22–23)	97 (50–143)	35 (25–44)	50 (44–55)
Dissection sharp/blunt	1.6:1	2.4:1	7.3:1	4.5:1	5:1
IPS score [5–8]	NA both anatomists were study evaluators	79%	49%	75%	62%
Joint entropy (speed/acceleration/direction)	9.15/9.17/3.29	7.29/7.31/3.25	NA. No colored gloves worn	8.55/8.62/3.36	8.47/8.63/3.40

Resident retention = data obtained 12 or 18 months after training. Values are shown as means with ranges in parentheses. Timing (ranges) in seconds and joint entropy measures compared with values of individual procedure score (IPS). NA = not available

### Detection of instrument use by different operators

Duration and type of instrument use also differentiated the three operator groups. Anatomists used Debakey forceps much of the time to spread tissue and relied on left- and right-handed blunt dissection. Anatomists also had greater duration for placement of the vessel loop and Army-Navy retractor than the surgeons. Expert surgeons had shorter duration of blunt dissection than residents but spent a greater proportion of their operating time using blunt dissection. Numbers of instrument changes also differentiated experts from other operators (Table 1).

## Computer vision entropy

Shannon joint entropy data shown as speed, acceleration and direction were least for experts (7.2/9/7.3) versus anatomists (9.4/9.5/3.3) and surgical residents (8.5/8.6/3.4) and aligned with individual procedure score, timing metrics and with levels of experience and training (Table 1). One left-handed expert surgeon showed greater acceleration and directional entropy change with dominant hand but same speed versus right hand, whereas all the right-handed surgeons showed higher speed with right versus left hands but similar acceleration and directional entropy.

## Discussion

Hand motion entropy differed between levels of training and was consistent with video-analysis-derived results and with individual procedure score, a benchmark of competence and a means to identify surgeons at all levels of training who are in need of remedial interventions [28]. The entropy finding conforms to the expectation of the dynamic systems theory of motor development which emphasizes a reduction in variability as part of the learning process that would be expected to differ as a result of training [29]. Our findings are also consistent with cognitive science research on motor learning, which shows higher levels of motor complexity in trainees than experts, because learned motor skills are associated with a decrease in movement complexity [30]. Entropy measures confirmed that lower bimanual entropy (equating to smoothness of hand movements with minimal wasted motion) is a reason why experienced and trained surgeons take less time to perform open surgical procedures.

The computer vision algorithm performed very well. It ‘locked onto’ the operator’s colored gloves and was not distracted by a different colored glove or when both of the operator’s (separately colored) gloves left the operating field and then one reentered. (See attached multimedia video.) However, we were unable to glean sufficient data on fine motor movements—carpal/metacarpal/digital—versus gross movement of the hand to use these analyses to explore in greater detail the basis of gestures associated with particular instruments or particular uses such as cutting, spreading and clamping or suturing gestures. Gesture recognition is captured in computer game users [12], these technologies might have potential for solving the problem of fine-motor analysis and advancing the precision surgical hand motion evaluations. In addition, devices such as Myoband® worn on the forearm can detect muscle movements associated with individual finger movements [31] and may assist trainee feedback. Gesture recognition and finger movements associated with specific instruments and procedural steps could be measured for many different surgical procedures. Frequency and timing of common surgical tasks such as skin incision, cut and spreading, retractor insertion, clamping and suturing could be monitored. Imbedding specialized motion detectors and identifiers into the surgical instruments themselves [32] would facilitate deconstruction and evaluation of the motions associated with a variety of procedures, not just the AA. Video recording with task analysis and hand motion entropy could be incorporated to make objective skill evaluations at all levels of surgical expertise. It could be integrated into routine residency operating-room training, provide experienced surgeons with valuable immediate feedback, quantitate inefficiencies (e.g. idle time, repeated instrument changes) and enable on-site mentored training to improve procedural skills, assist teaching and minimize the reinforcement of errors in procedural steps.

## Expert performance

Expert performance was characterized by a minimal amount of idle time and the lowest number of instrument changes during the procedure. Experts’ calculated joint entropy was least among all the operators. The idle time and instrument-change metrics are technical assessments that can easily be targeted for continuing education/skills-improvement for individual surgeons and so may represent a valuable training tool. Although joint entropy will be an unfamiliar concept to many, our data show that overall efficiency and smoothness of motion are captured by this metric, and that it is therefore an important contribution to targeted skills acquisition and maintenance.

## Potential benefits for surgical training programs

Both computer programs used for this work are available for free: Shannon entropy (https://biomedical-engineering-online.biomedcentral.com/articles/10.1186/s12938-019-0650-5) and computer vision algorithms (OpenCV Python library, Copyright 2013, Alexander Mordvintsev & Abid K. Revision). High definition video can be collected using consumer level digital cameras costing $1200 or less. As noted above, addition of detectors on instruments [32] could avoid burdensome collection of timing data and enable other benefits such as timing of start and end of surgery and implementation of novel performance assessments. Interval measurements of surgeons performing eligible procedures in a training program could be a means of assessing resident performance and identification of need for individualized remediation. Remediation plans could be tailored to discrete steps of the procedure and range anywhere from reviewing anatomy to repeating individual procedures or tasks in a mentored fashion on a cadaver or a simulator. Use of Shannon entropy with procedural video-analysis could provide objective evaluation of sub-tasks in a procedure such as knot tying and suturing, and assist surgical quality assurance programs or training course evaluations. With routine use in training programs of differentially colored gloves and video recording of the surgeon’s hands and the operative site of specific procedures, computer vision algorithms would be an objective means of evaluating residents. Tools and consent for video recording and analysis in health care settings are summarized elsewhere [33]. These evaluations are inexpensive, might have applicability in both sophisticated surgical programs and in countries where resources and mentors time-availability are limited.

## Limitations

Video acquisition requires that the angle and distance of the camera from the operative site be standardized if comparative measures are to be made in the same individuals. The collection of entropy data will not be valid if the hands cannot be visualized (e.g., intra-abdominal procedures) or when the operative field is obscured for any reason (e.g., operating lights). The study experimental design was based on a convenience sample of videos and participants, so that only proof-of-concept examples were obtained from each of the categories of operators. A prospective study confirming data collection and analysis methods and their relation to surgical performance is needed.

## Conclusion

In this proof-of-concept study, we show that Shannon joint entropy analysis, number of instrument changes, total time and ratios of idle to active and blunt and sharp dissection times as a proportion of total procedure time, can be used to discriminate usefully between expert and non-expert surgeons and non-clinicians, demonstrating the effects of training on hand motion. Hand motion metrics were congruent with a labor-intensive validated procedure performance score, suggesting that video recording with automated Shannon entropy measures can contribute to the range of modalities available to educators, training course designers, and surgical quality assurance programs and be helpful to surgical trainees by providing feedback of intra-operative technical skills.

## Supplementary Information

Below is the link to the electronic supplementary material.

Multimedia video (see separate file) shows the algorithm “lock onto” the surgeon’s hands, despite other hands coming into the field with different colored gloves, the algorithm is not distracted. The computer vision algorithm “re-attaches” onto the surgeon’s hands immediately when both of the operator’s (separately colored) gloves leave the operating field and then one reenters. Supplementary file1 (MP4 18.3 Mb)
